# Is Simulation of Glued Contact Sufficient to Simulate Nonlinear Failure Behaviour in Dental Shear Bond Strength Tests?

**DOI:** 10.1016/j.identj.2025.03.007

**Published:** 2025-04-05

**Authors:** Ahmed M. Ismail, Ahmed ElBanna, Tamer M. Nassef, Ludger Keilig, Christoph Bourauel

**Affiliations:** a Oral Technology, University Hospital Bonn, Bonn, Germany; b Biomaterials Department, Faculty of Dentistry, Ain Shams University, Cairo, Egypt; c Computer and Software Engineering Department, Misr University for Science and Technology, Cairo, Egypt; d Department of Prosthodontics, Preclinical Education and Dental Materials Science, School of Dentistry, University Hospital Bonn, Bonn, Germany

**Keywords:** Finite element analysis, Non-linear failure analysis, Dental adhesive, Dental shear bond strength, Micro shear bond strength

## Abstract

The aim of this study was to develop a numerical model for simulating shear bond strength tests with different specimen sizes and loading techniques. A finite element model was generated consisting of a composite specimen bonded to dentin substrate surrounded by enamel, acrylic resin and polypropylene tube. Four models were created simulating macro (diameter 1.8 mm) and micro (0.8 mm) sized specimens loaded by either a chisel or a wire loop. Experimental data from a previously published study using the identical specimen diameter and shearing tools were used as reference. Four groups were established: macro shear wire loop (group 1), micro shear wire loop (group 2), macro shear chisel (group 3), and micro shear chisel (group 4). In the simulations, contact-based glue failure based on shear contact stresses (series 1) or a combination of shear and normal contact stresses (series 2) were used to simulate the progressive failure of the specimens. Shear and normal failure stress limits were fitted to the experimental results in sensitivity analyses by varying both stresses. Experimental failure forces could be reproduced using group-specific shear stress limits of 71 (group 1), 48 (group 2), 106 (group 3), and 131 MPa (group 4) in series 1. However, when also considering normal stresses, no single, unique pair of shear and normal failure stresses can lead to the experimental failure force values for all groups. In conclusion, no unique pair of shear and normal stresses can provide the same failure force values for different shear setup geometries.

## Introduction

Owing to the simplicity of testing procedures, shear bond strength (SBS) tests are considered the most commonly used methods for bond strength measurement.^1^ Advantages of shear tests include the easiness of specimen preparation, simple testing protocol, lower incidence of pretest failure, and minimal equipment needed. Knife-edged chisel was the traditional loading method despite having a lot of concerns regarding stress concentration at a specific point on the bonded interface leading to complex representation of stresses and underestimated bond strength value. As an alternative, the wire loop method has been used to de-bond specimens in SBS tests.^2^ Classically, these test methods calculate the shear bond strength of a specimen as force at failure divided by the original contact area.^3^, ^4^ Typically, manufacturers of dental adhesives list some values for the shear bond strength of their materials in the corresponding material data sheets but not necessarily give further information on how these values were determined. However, previous studies have shown that variations in the test parameters can influence the determined values over a larger range.^5^, ^6^, ^7^

In bond strength studies, finite element analysis (FEA) is a useful tool for analysing generated stresses at the bonding interface with a particularly applied load either in shear or tensile modes of testing.^3^^,^^8^ The results of the FEA depend on its modelling methodology and the values assigned to basic material properties (eg, elastic or Young's modulus and Poisson's ratio), material limits (eg, plasticity or yield limit), or contact-related parameters (eg, coefficient of friction, failure strength of a connection) besides many other factors.^9^ For an accurate simulation, the relevant material parameters need to be known and included in the simulations.

Jin et al. have worked on simulating bond strength tests using FEA and came to a conclusion that none of the bond strength tests could give solely ‘shear’ or ‘tensile’ stresses, and that there are always complex stress distributions that adversely affect the reliability of bond strength test results.^3^ Ereifej et al. reported in 2011 that while there are extensive discussions of the standardisation of SBS tests, there are also difficulties in agreeing on a specific test setup.^10^ Since then the ISO standard 29022 was published, there has been continuous discussion about the optimisation of shear-testing methodology and the applied shearing tools.

We thus performed finite element simulations and sensitivity analyses to explore the relationships between different test geometries and the influence of shear and normal stress on failure forces. Our aim was to establish a simplified numerical model based on glued contact modelling that allows us to study the shear bond strength tests systematically, and to investigate the influence of specimen diameter and shearing tool on the determined shear bond strength values.

## Materials and Methods

**Fig 1:**
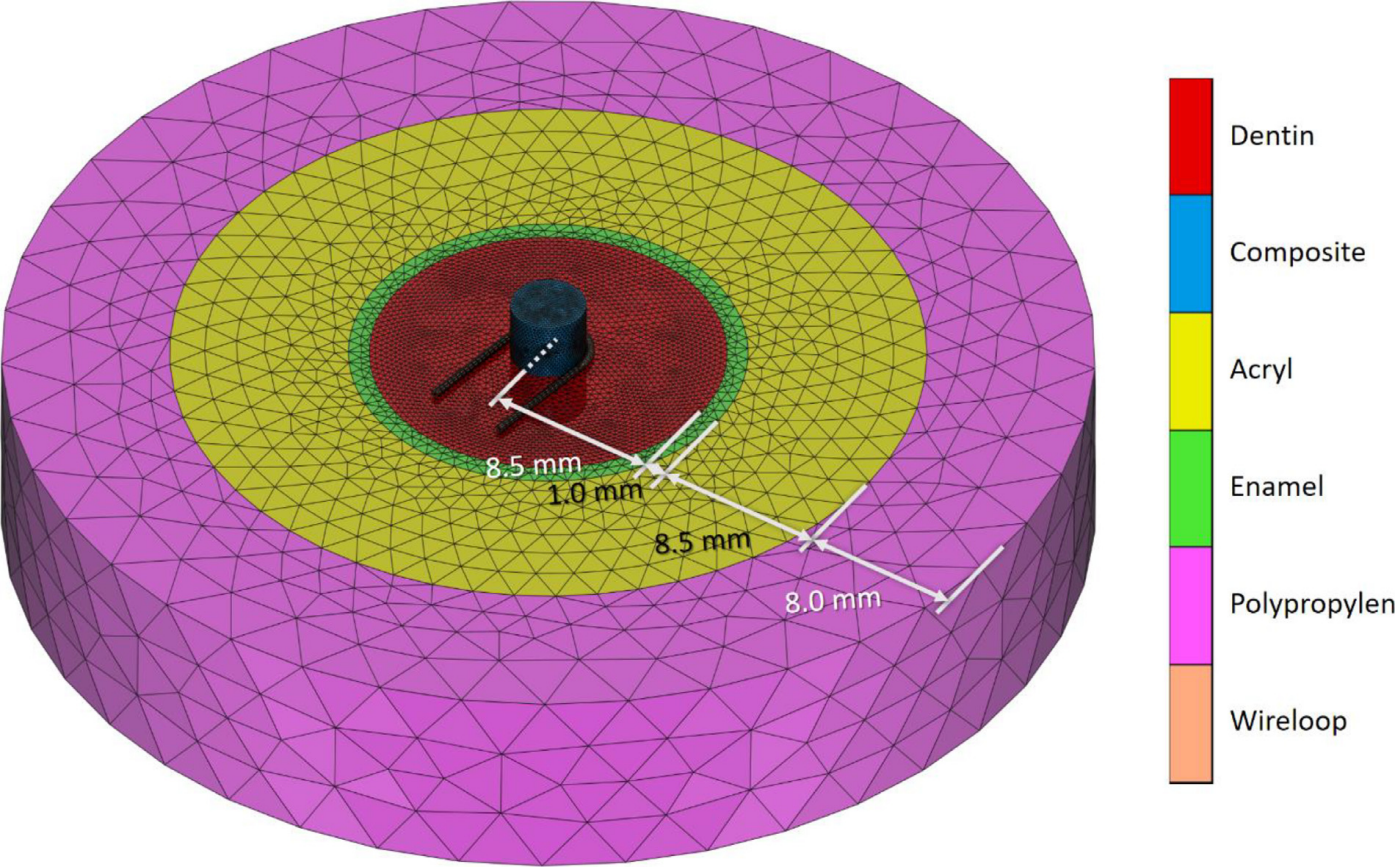
Schematic representation of model geometries. Components are identified in the coloured legend.

Experimental models were established as presented in a previously published experimental study.^7^ In the current study, a commercial FE software^11^ was used to create the virtual 3-dimensional model corresponding to the experimental situation. The numerical model was created using the same dimensions and material properties of the in vitro experiment. The FE model for shear testing was modelled using 6 different components (see Figure 1): composite cylinder, dentin cylinder, enamel tube, acrylic tube, polypropylene tube, and the shearing tool. The adhesive layer was not modelled directly; instead, the bond between composite specimen and dentin body was realised using a ‘glued’ contact condition with potential failure between these 2 components (see below).

Four main models were created, differing in the specimen diameter (1.8 mm [Macro] and 0.8 mm [Micro]) and the shearing tools (chisel [CH] and wire loop [WL]). The chisel was modelled as a cylindrical surface (rigid geometry) with a diameter of 0.3 mm, as determined on the chisel used in the experiments. The wire was modelled as deformable volume model with a diameter of 0.2 mm, again based on the experimental conditions.

Material properties (elastic modulus and Poisson's ratio) were taken from literature (see Table 1). A standardised Poisson's ratio of 0.3 was used for all materials instead of, for example, 0.31 for dentin^12^ or 0.28 for steel,^3^ based on the results of a sensitivity analysis, showing that minor variations of around 10% had very limited influence on the results. To simplify the numerical model, the anisotropic material behaviour of dentin was not considered. All enlisted materials were assumed to be isotropic, homogeneous, and with linear elastic behaviour. Linear (4-noded) tetrahedral elements were used for all model components except for the chisel, which was—as stated above—modelled with a rigid cylindrical surface. A sensitivity analysis was performed to select the best element size for each modelled material to obtain accurate results with reasonable processing time. Table 2 shows the details of finite element model structures, including the finally chosen element sizes.

**Table 1:** Material properties used in this finite element study.

Material	Elastic modulus (MPa)	Poisson's ratio
Composite (private communication with the manufacturer)	9000	0.3
Dentin^12^	17,000	0.3
Enamel^12^	41,000	0.3
Acryl^13^	2500	0.3
Polypropylene^14^	900	0.3
Wire loop (orthodontic ligature wire from stainless steel)^3^	210,000	0.3

**Table 2:** Details of the meshing parameters used to generate tetrahedral finite element models.

Material	No. of elements	No. of nodes	Smallest element edge size (mm)	Largest element edge size (mm)
Composite (macro shear)	118,930	21,614	0.0125	0.1
Composite (micro shear)	24,318	4718	0.0125	0.1
Dentin	64,602	12,664	0.2	0.8
Enamel	7079	2178	0.4	1
Acryl	8141	2085	1	2
Polypropylene	3596	981	2	2
Wire loop (macro)	10,119	2605	0.03	0.15
Wire loop (micro)	9982	2532	0.03	0.125

Specimen loading with CH and WL was simulated by pushing the shearing tool parallel to the dentin surface against the composite specimen. In all cases the shearing tool was placed directly in contact to the dentin surface. The rounded cross section of the shearing tools results in a contact point at the specimen 0.15 mm and 0.10 mm above the dentin surface for the CH and WL, respectively. This results in different loading points for both tools but reflects the experimental conditions correctly. As it was done in the experiments, the forces were determined by measuring the force acting on the (simulated) shearing tool.

Circumferential nodal constraints in X, Y, and Z directions were applied to polypropylene outer nodes as a boundary condition to simulate the attachment grips of the material testing machine. Displacement of the shearing tools CH and WL against the composite specimens was increased linearly with time. All simulations were performed as dynamic simulations to avoid singularities after complete separation of the composite bodies from the dentin base. As an idealisation we assumed a constant density of 1 g/mm^3^ for all materials. Preliminary test simulations with variation of the density by a factor of 10 showed no influence on the specimen behaviour during the adhesive failure itself.

### Numerical simulation of the de-bonding process

Simulating the initiation and propagation of cracks in a FE model is an exhausting task. It requires carefully choosing several parameters that describe the material failure process that leads to the initiation of a crack, and all these parameters need to be verified. This holds especially if more than 1 material with different failure characteristics is involved. We therefore decided to choose a different, simplified approach to determine the failure of the bond between composite and dentin, using only the numerical modelling of the contact between the involved components. Technically speaking, this approach only allows to simulate pure adhesive failure, but we wanted to check whether this approach would catch the behaviour for all SBS tests independent of their failure mode.

The failure behaviour of the adhesive connection was simulated using the glue failure feature built into MSC Marc/Mentat (termed ‘glue breaking’ in the documentation of that software package). This allows the change of the defined contact interaction between composite body and dentin substrate from glued to touching (ie, failed) once a specific failure threshold is reached. During the simulation, the following stress-based criteria were used to determine if the glued connection has failed at a node:

$${\left(\frac{\sigma_{n}}{S_{n}}\right)}^{2}+{\left(\frac{\sigma_{t}}{S_{t}}\right)}^{2}>1$$

Here *σ_n_* is the contact normal stress (in tension only), *σ_t_* is the contact tangential stress, *S_n_* is the contact normal stress (CNS) limits, and *S_t_* are the contact tangential stress (CTS) limits (MSC Software Corporation, 2020). The software allows to ignore the influence of either CNS or CTS completely—in other words, to assume that either *S_n_ = ∞* or *S_t_ = ∞*, or to use both CNS and CTS together at the same time.

In those regions where glue failure fracture has been detected, separation and friction between the contact partners are permitted. It must be stated that this is a local material-dependent criterion, working on the nodal stress levels. This contrasts with the shear bond strength values determined in experimental tests, where the total original contact area is considered in the formula of stress calculation. The CNS and CTS limits of a glued connection are material dependent and unknown. While the experimentally determined shear bond strength of an adhesive might be a good starting point for parameter identification, the actual values for the CNS and CTS limits might differ from the value of the SBS. To establish a numerical model for the SBS tests, we therefore need to determine the corresponding CNS and CTS limits for the adhesive used in the previous experimental study. As this glue failure feature works on local region (ie, on each triangle in contact separately), we assumed that these stress limits were independent from the global test parameters specimen diameter and shearing tool.

Adhesive failure is often considered as driven primarily by shearing, as can be seen, for example, in the working instructions for de-bonding orthodontic brackets^15^, ^16^ Therefore, we used 2 different approaches. In the first part of our study we assumed that shear test is primarily driven by shear stresses in the adhesive layer, and that the normal stresses in this layer do not contribute to the failure (ie, the CNS limit *S_n_* is infinite). In the second part we used both normal and shear stresses at the same time to determine adhesive failure.

### Adhesive failure simulated with shear stress only

In the first series of simulations we only considered the CTS limit to trigger an adhesive failure. The CNS was not considered—in other words, the CNS limit was assumed to be infinite. Using the FE models described above we varied the value for the CTS limit as simulation input to determine the resulting failure force as a function of this CTS limit. Mean failure force and standard deviation (SD) from the previous experimental study were used as targets (see Table 3 for a summary of these data). Data from all specimens from that study were used, independent of the failure mode observed for the specimens.

**Table 3:** Experimental failure force values, failure force standard deviations, and corresponding CTS limits for each geometry, as reported in Ismail et al.^7^ These values were used as targets for the current parameter identification.

Geometry	Mean failure force (N) in experiments	Standard deviation (N)	Determined shear bond strength (MPa)
Macro-WL	46.0	10.7	71
Micro-WL	9.3	4.3	48
Macro-CH	64.9	18.8	106
Micro-CH	27.0	7.1	131

The manufacturer's shear bond strength value for the adhesive (38 MPa) was used as a starting value for the CTS limit, then it was systematically varied until the mean value of the experimentally determined failure force for the corresponding specimen diameter and shearing tool was obtained. The same procedure was used to determine the CTS limits resulting in a failure force of (mean + SD) and (mean − SD). This process was repeated with the FE models for both diameters and both shearing tools. After identifying the CTS limits corresponding to the listed experimental values, additional simulations were performed with small variations applied to the limit to ensure smoother graph generation. The minimum resolution for the stress limits was 1 MPa. No parameter variation below this resolution was performed.

### Adhesive failure simulated based on combined shear and normal stress

In the second series of simulations we considered both CNS and CTS limits to trigger an adhesive failure. The normal component of stresses inevitably exists during shear testing owing to the obligatory offset for both loading devices (0.1 mm for WL and 0.15 for CH) represented by the radius of separation from the substrate (dentin). Thus, CNS and CTS limits were provided as simulation input to determine the resulting failure force as a function of input parameters. The first series of simulations, as described in the previous section, showed that the numerically determined CTS limits differed clearly from the manufacturer-provided SBS value. Therefore, the values of the CTS limits determined in the first series were used as starting points for both CNS and CTS limits in the second series.

We assumed that there is no single unique pair of CNS and CTS limits that results in our desired failure force, but instead a large set of such pairs resulting in the same force. Ideally, these data points would follow a continuous curve in the 2-dimensional space spanned by the input parameters. Therefore, we decided on the following systematic approach to identify the correct parameter sets for both limits. We started by setting both CNS and CTS limits to multiples (1.5X, 2X, 2.5X, and 3X) of the CTS limit values determined in the first series as described in the previous section. For each of the listed multiples 2 new subseries were started. In 1 subseries the CNS limit was fixed and the CTS limit was varied as before until experimental mean, mean − SD, and mean + SD failure forces were reached. In the other subseries the same was done with fixed CTS limit and varied CNS limit. Again, the simulation series were supplemented with additional simulations with minor variations of the stress limit values to smoothen the created graphs. A minimum resolution of 1 MPa was used in these parameter variations, same as in the previous series.

## Results

### Comparison of the numerical simulation with the experimental behaviour

**Fig 2:**
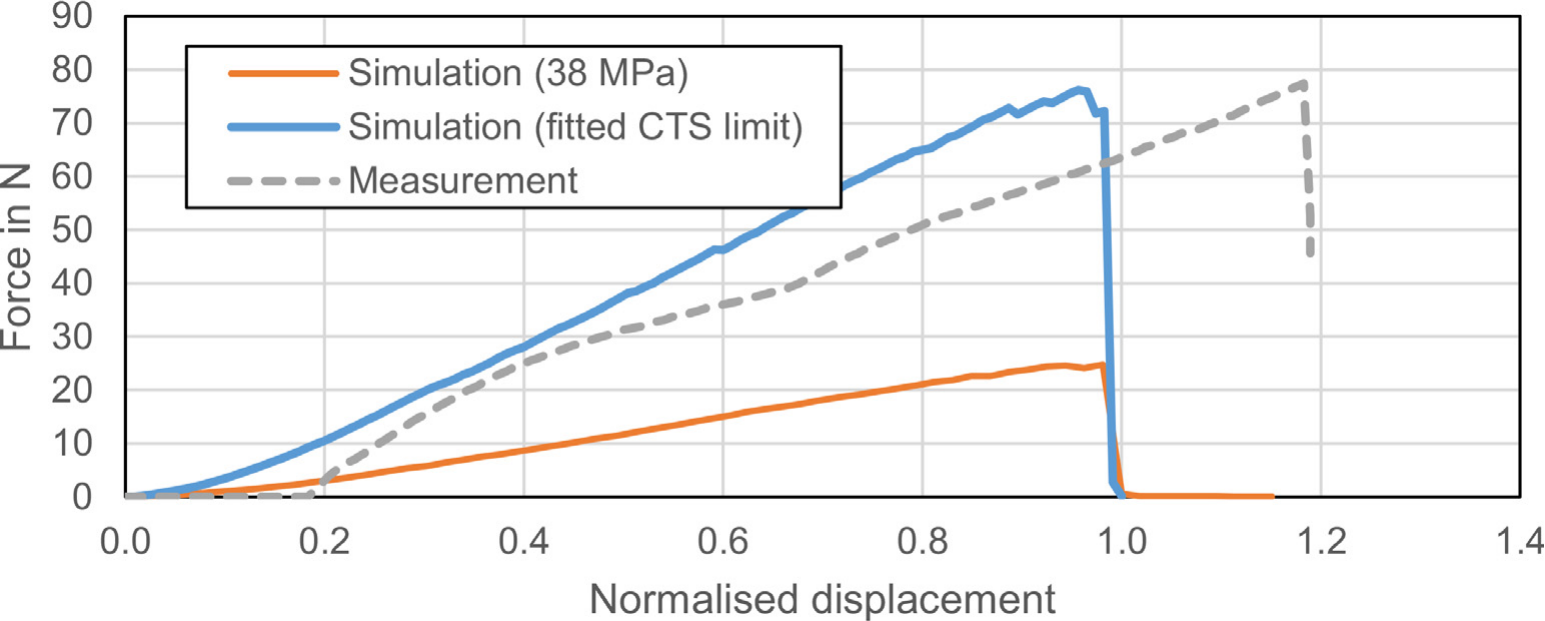
Comparison of experimental and numerical force-displacement curves for the Macro-CH group. The x-axis is normalised in such a way that the displacement between first load application and failure is 1. The slight offset at the beginning of the experimental curve is due to a small gap between the shearing tool and the composite body when starting data recording for that measurement. Both numerical (solid) curves show the typical course of ‘load until failure’ curves. With the manufacturer-supplied SBS value, the simulation did not reach the experimentally determined force. The numerical curve after parameter fitting (solid blue) is in good correlation with experimental results (dotted grey).

To verify whether the numerical simulation correctly describes the experimentally observed failure behaviour, we compared the course of the force-displacement curves registered during the experiments with those of the simulations. Figure 2 shows a comparison between the experimentally determined force-displacement curve of one of the Macro-CH specimens with 2 numerically determined curves for the same geometry which used only the CTS limit to determine adhesive failure. The x-axis in Figure 2 is normalised in such a way that the displacement between first contact and failure is 1 (in arbitrary units). This normalisation was necessary, as the simulation does not reproduce the elasticity and play within the frame of the materials testing machine. One numerical curve uses the manufacturer's shear bond strength of 38 MPa as CTS limit, the second numerical curve uses a CTS limit of 106 MPa fitted to this geometry.

**Fig 3:**
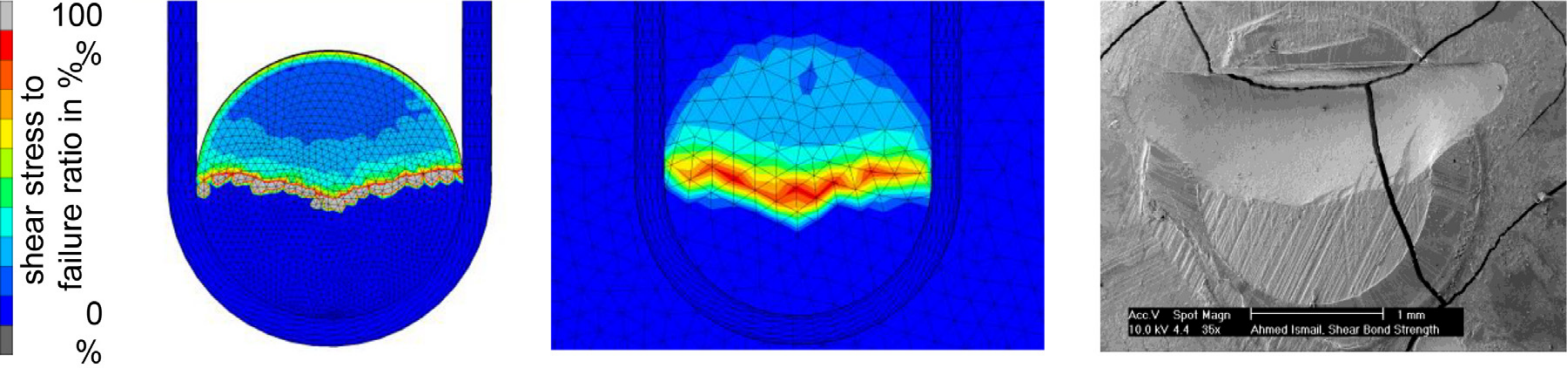
Comparison between adhesive failure progress in the numerical simulation and in the experimental study. Progress of the adhesive failure in the simulation for the Macro-WL geometry on the bottom (bonded) side of the composite specimen (left) and the top side of the dentin base (middle). Colours indicate the stress value relative to the failure threshold. The lower half is already de-bonded, indicated by a ratio of 0% (corresponding to a shear stress of 0 MPa). Surface analysis of a Macro-WL dentin sample with scanning electron microscopy after a mixed mode failure (adhesive and cohesive in dentin) in vitro showed a pattern similar to progressing failure of the interface observed in the simulations (right).

Scanning electron microscopy (SEM) images of the dentin substrate after de-bonding were available from the experimental part. We compared these images with the stress and failure patterns from the corresponding simulations within the same groups. Figure 3 shows the distribution of the contact tangential stresses in the contact interface between composite specimen and dentin base for a simulation with the Macro-WL geometry. The right-most image shows a SEM image of a dentin sample surface of a corresponding test specimen from the experimental study after de-bonding as a comparison.

### Results of the first simulation series with CTS only

**Fig 4:**
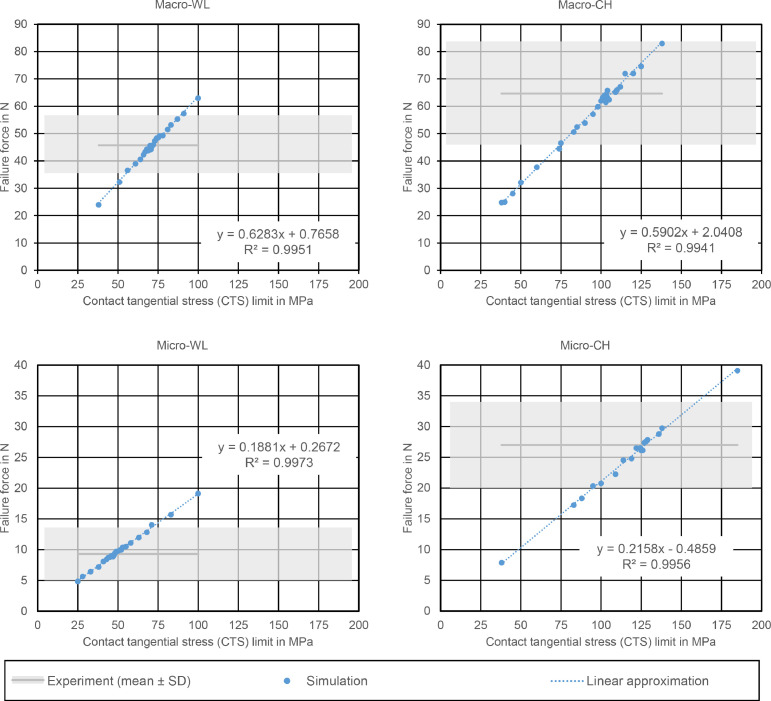
Simulation results for the 4 geometries. For all subfigures, each blue dot represents 1 simulation. The dashed line is a linear trend based on the results; the corresponding formula is given as well. The grey region represents the mean failure force ± SD from the experiment.

**Fig 5:**
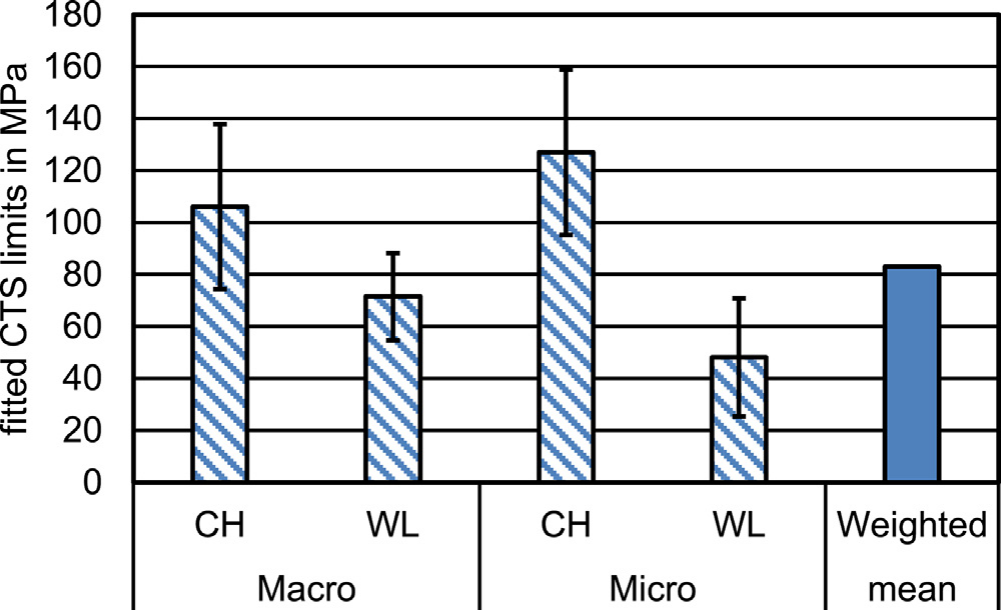
Determined CTS limits and ranges for the 4 different groups. The height of the bars corresponds to that CTS limit that resulted in the experimentally determined mean failure force, while the whiskers represent the limits resulting in (mean + SD) and (mean − SD) failure force.

In all groups, using the manufacturer-supplied value for the SBS did not result in a failure force corresponding to the experimental values. Figure 4 shows the results for all 4 groups that have been calculated in our first simulation series, using only shear stress to determine adhesive failure. In all subfigures, the experimentally determined mean ± SD values for the failure forces are indicated with a horizontal grey band. We used this band to indicate the experimental data, as there is no directly corresponding parameter in the experiment to be used as a parameter on the x-axis. The relationship between the configured CTS limit and the resulting failure force was linear, with a R^2^ above 0.99 for all groups. The intersection of the trend line with the grey band describing the experimental failure force yields the CTS limits required to achieve the corresponding failure force. This resulted in fitted CTS limit ranges of 106 (±32) MPa, 72 (±17) MPa, 127 (±32) MPa, and 48 (±28) MPa for Macro-CH, Macro-WL, Micro-CH, and Micro-WL, respectively (see Figure 5).

**Fig 6:**
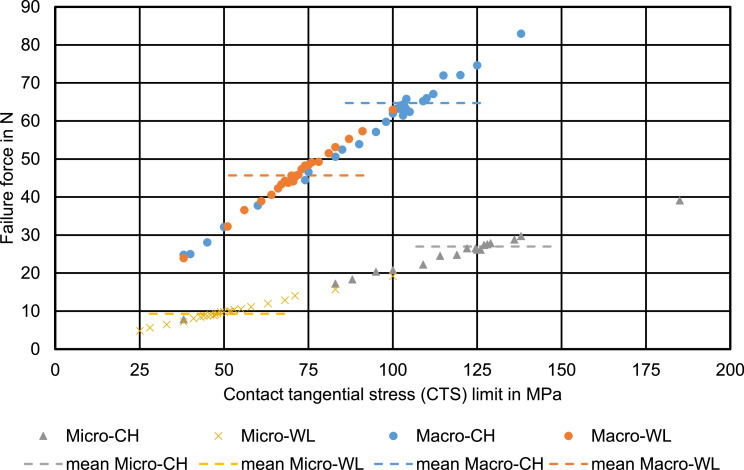
Direct comparison of the fitting curves for all 4 groups. The dashed lines represent the mean failure force taken from the experimental study for the corresponding groups.

For better comparison between the groups, the 4 fitting curves were additionally collected in Figure 6. It is clearly visible that the 2 shearing tools used with identical specimen diameter resulted in similar fitting curves, while the curves for the Macro specimens differed greatly from the curves for the Micro specimens. This is also visible in the formulas for the regression curves in Figure 4.

**Fig 7:**
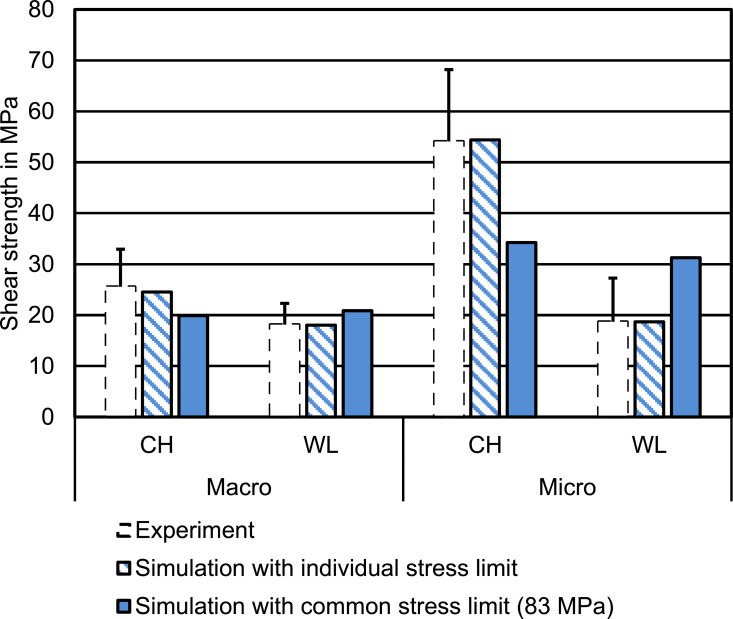
Determination of the shear bond strength as failure force divided by total interface area, according to experimental protocol. Given that the individual stress limits are fitted in such a way that the FE models showed the same failure force as their corresponding experimental counterparts, and the dimensions are identical, the resulting shear bond strength per group with individual stress limits is identical by design. Using a common stress limit between the groups, the resulting simulated shear bond strength diverges from the experimental values, especially for the Micro geometry.

Since there was no common overlap between the CTS limit ranges presented above, we additionally determined the weighted mean value (using the 1/SD as the weight) of the above ranges and ran additional simulations with this value for all 4 groups. In Figure 7 a comparison of the shear bond strength from the experiments (calculated as failure force divided by the original total surface area) with the values determined in the simulations using individually fitted CTS limits (which must be identical, because force as well as specimen dimension are identical) and with the values obtained using the common CTS limit of 83 MPa (weighted mean) is presented.

### Results of the second simulation series with CTS and CNS

**Fig 8:**
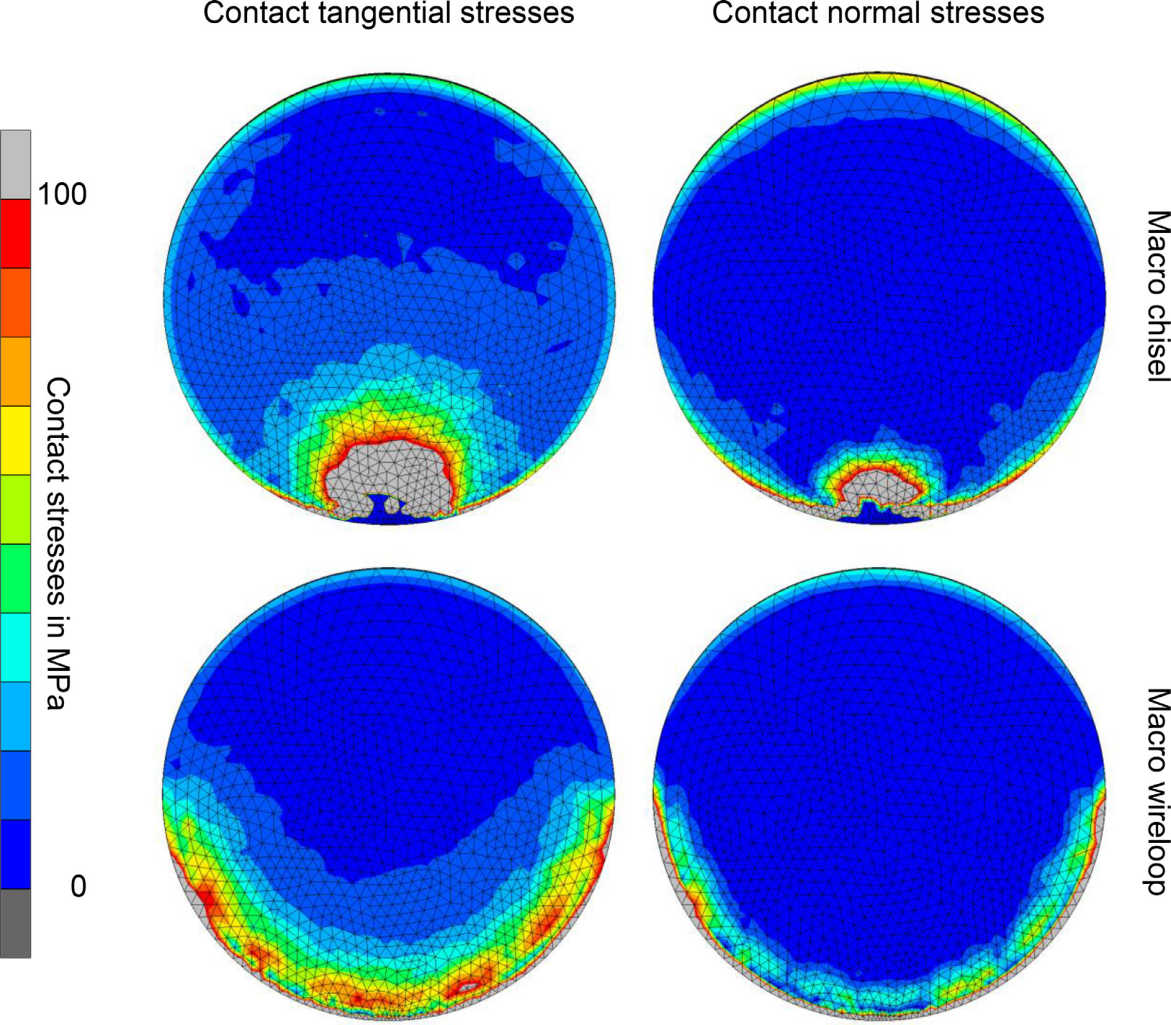
Distribution of shear (left) and normal (right) stresses in the adhesive interface between the bonded components during loading with the chisel (top row) and the wire loop (bottom row) in the Macro geometry. Regions of the highest stresses correspond to the position of the loading tools. Load direction is upwards in the images. Specimens were loaded with the same force that caused specimen failure in the experiment. Configured stress limits were 400 MPa for both the CTS and CNS limit. In some regions, normal stresses reach approximately 50% of the shear stresses.

To judge the amount of normal stresses and the ratio between normal and shear stresses introduced into the adhesive layer during our shear tests, Figure 8 shows the distribution of these stresses in the interface between the bonded components as an example for the Macro-WL and the Macro-CH geometries. In those simulations, the specimens were loaded with the same force that caused de-bonding under experimental conditions, but both CTS and CNS limits were each set to 400 MPa to avoid early failure and thus allow to see how shear and normal stresses develop. While the presented experimental setup for this kind of shear bond strength test is designed to load the bonded components mainly in a shear direction, the normal loads still appear in a relevant magnitude. In several regions, the normal stresses reach 50% of the amount of shear stresses. This demonstrates that the strength of the adhesive in tensile direction might influence the results of such tests.

**Fig 9:**
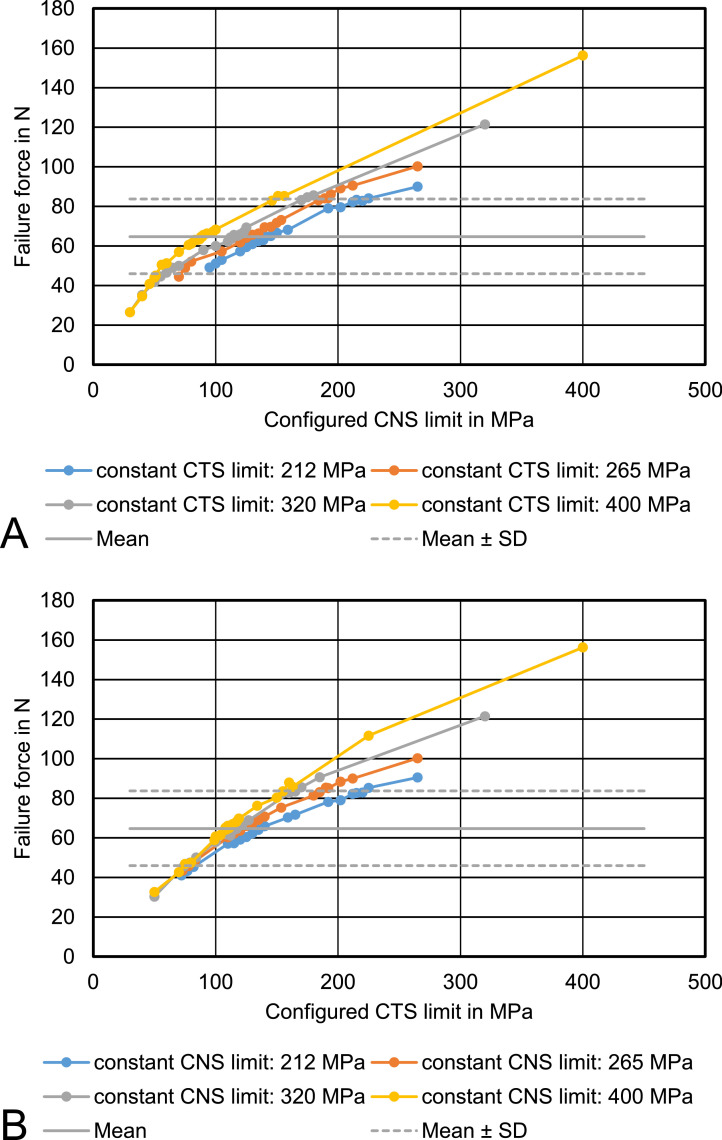
Determined failure force in some subseries determined for the Macro-CH geometry. In A, the CTS limit is kept fixed and the CNS limit is varied; in B, the CNS limit is kept fixed and the CTS limit is varied. The experimental mean value and the standard deviation of the failure force are indicated by the solid and dotted grey lines, respectively.

Figure 9 shows selected data curves from the Macro-CH geometry for the subseries that kept 1 stress limit fixed (either CTS or CNS; see Figure 9A and 9B, respectively) and varied the other limit. Contrary to the previous simulation series, there was no linear relationship between the configured stress limits and the resulting failure force.

**Fig 10:**
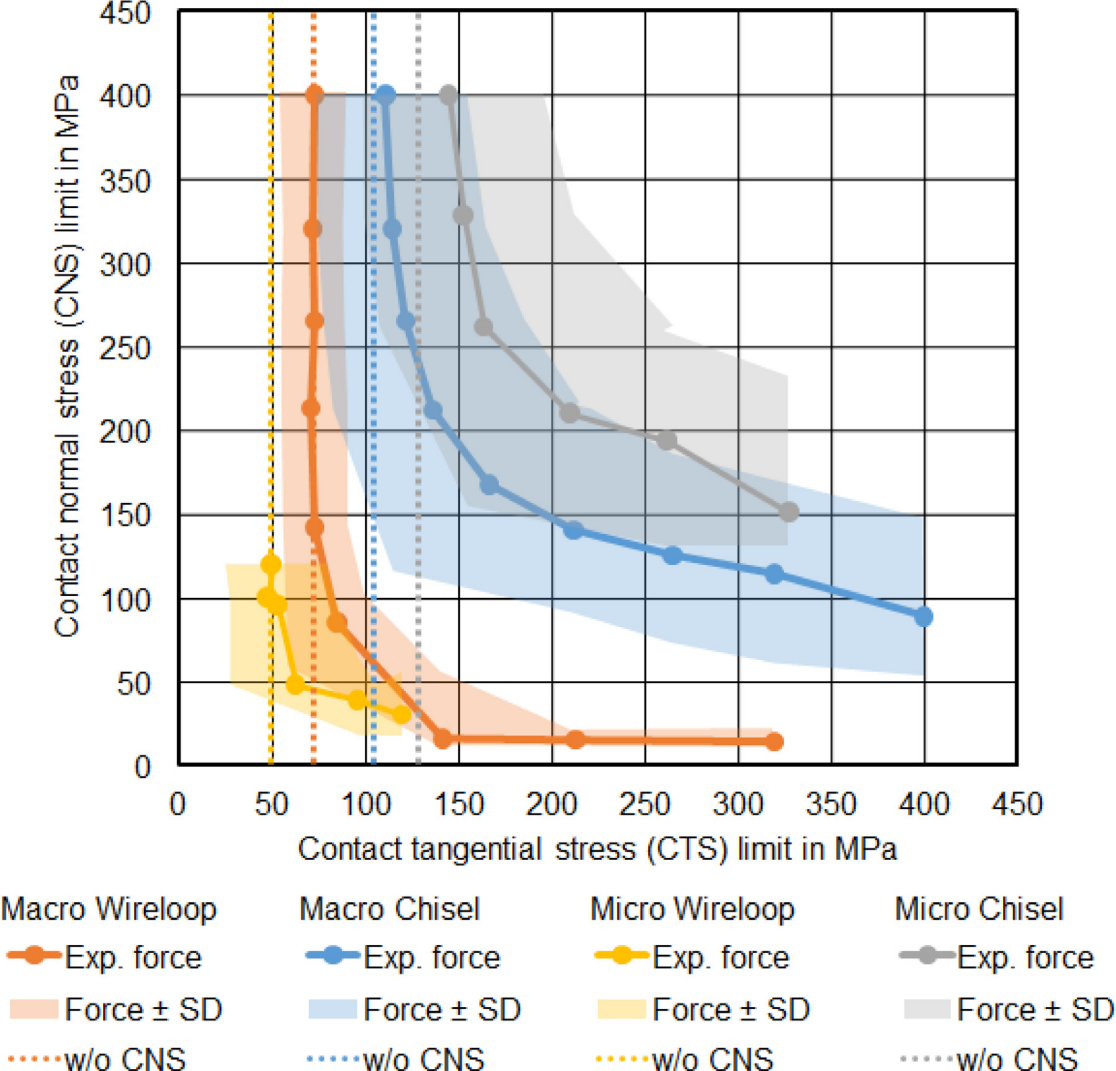
Visualisation of the parameter space for the CTS and CNS limit variation. Each coloured dot (labelled ‘Exp. force’) corresponds to a parameter pair that resulted in a simulation with the experimentally determined mean force of the corresponding geometry. The coloured bands (‘Force ± SD’) show the region in which the simulated force was within the range of experimental mean force plus or minus standard deviation. The vertical dotted lines (‘w/o CNS’) indicate the results of the previous series that ignored the contact normal stresses for the failure contribution.

Analysis of these subseries allowed to identify for each geometry separately matching pairs of CTS and CNS limits that, when used in the simulations, resulted in the same failure force as observed in the experiments. The same approach was used to determine upper and lower bounds for the limit pairs in which the simulation resulted in a failure force within the range of experimental mean force value plus or minus standard deviation. The resulting limit pair ranges are visualised in Figure 10, using different colours for all investigated geometries. In that figure, each dot corresponds to 1 CTS and CNS limit pair that resulted in a simulation with the same failure force equal to the experimental mean failure force for the same geometry. CTS and CNS limit pairs within the coloured bands result in a failure force within the range of experimental mean force ± SD for the corresponding geometry. As reference, the results of the first part of the study (which assumed an infinite CNS limit) are indicated with dotted vertical thin lines.

From Figure 10 it seems obvious that there is no single, unique CTS and CNS limit pair that results in the experimentally determined failure forces that is valid for all 4 studied geometries. Additionally, when observing the course of the graphs for increasing CNS limits, it seems obvious to predict that the graphs converge to that value determined in the first simulation series for each geometry separately.

## Discussion

Previous studies have shown that methodological variations allowed in the typical shear bond strength test in dental adhesives influence the magnitude of the determined bond strength values.^6^^,^^7^^,^^17^^,^^18^ A numerical model of such a SBS test setup would allow to investigate the influence of different test parameters on the resulting SBS values, and in turn would help to compare the SBS values of different adhesives obtained under varying test conditions. Such different test parameters would include controlled variations, like the specimen size (diameter) or the shearing tool, as well as uncontrolled parameters, like an imprecise positioning of the shearing tool. While Jin at al. simulated the stresses during different types of SBS tests,^3^ they did not simulate the progressing failure between the bonded surfaces. It should be noted that, until now, there has been no published numerical study reporting on SBS tests that include the failure of the adhesive, and therefore we decided to develop a model ourselves. We have chosen our previous experimental study as data source for numerical model development, as our detailed first-hand knowledge of the experimental environment allowed us to reproduce it fully in the numerical model.

The FE model was generated with dimensions identical to the experimental model to be able to use the experimental data for the parameter identification in the developed numerical model. The rather simple geometry used in the experimental shear bond strength tests allowed for a quick generation of a corresponding FE model for all 4 groups. Experimental data, specifically mean stress and mean force at failure values for each study group, from the previous study were used as input.^7^

Type and position of the shearing tools in the simulations were chosen according to the experimental conditions. As in the experiment, the shearing tools in the simulations were positioned directly on top of the dentin surface. Due to the geometry of the shearing tools, the resulting point of load application at the composite specimen is at an offset that is equivalent to the radii of the WL and the chisel tip circular cross sections. This offset is 0.10 mm and 0.15 mm for the WL and the CH, respectively. The difference in the vertical position of the point of force application can lead to some bending of the composite specimen, influence the ratio between the amount of shear and normal stresses between the bonded components, and therefore change the failure behaviour of the adhesive. Nonetheless, the simulations replicate the experimental situation, and any changes in the ratio between shear and normal stresses in the experiment should be reflected in the same way in the simulation.

We simulated the adhesive connection using the glue failure feature of our FE software. Therefore, there was no model component representing the adhesive layer. Instead, the adhesive was represented using the numerical parameters of the 2 stress limits for CTS and CNS. This does not represent the experimental situation fully, but this idealisation was necessary. Modelling the adhesive layer with solid elements would have required very small elements due to the low thickness of the adhesive layer. For example, in the numerical study of Jin et al., a layer thickness of 5 µm was assumed,^3^ and a more recent paper from Tang et al. measured the adhesive layer thickness down to 12.8 µm depending on the application protocol for the adhesive.^19^ Using such a low layer thickness and subsequent large number of elements would have resulted in a steep increase of the computation time. In addition, simulating the crack initiation and propagation requires a lot of finely tuned numerical parameters to describe the cracking process in a realistic way. In this study, this would have required to simulate the crack propagation through the adhesive as well as potentially through the dentin base and/or the composite test specimen. Unfortunately, there is not enough information in the literature to model this crack propagation correctly, and our existing experimental data was not sufficient to determine the required parameters. This holds especially given that such crack propagation data would be required for the dentin and the composite body on top of the parameters of the adhesive, which were the main focus of our study. It has to be stated, therefore, that the presented method only allows to model a purely adhesive failure and cannot model a mixed failure that was seen for part of the specimens in the previous experimental study.

The direct comparison between the experiments and the numerical simulations is promising. The simulations showed a developing failure pattern in the bonded interface which matched the experimental behaviour documented in SEM pictures from our own study as well as in studies from other groups.^10^^,^^20^, ^21^, ^22^ In the same way, our numerically determined force/deflection curves showed a similar course as typically observed in such studies.^10^^,^^20^, ^21^, ^22^ In our simulations, the displacement until failure was typically smaller than in the experiment. This can be explained by a possible micro movments among the experimental components as well as elastic deformations within the complex measurement setup on the experimental side. These were not present in the simulation, as the load was directly applied to the shearing tool. It can be argued that the missing layer of (elastic) adhesive artificially decreased the mobility of our specimens, but if we consider the typical thickness of this layer, we are sure that the effect of the (missing) layer vanishes against the other elastic effects of the material testing machine outside of the simulated model.

SBS tests are designed to maximise the shear loading of the adhesive while at the same time reducing the normal load. Still, this loading method does not completely avoid normal stresses in the adhesive,^3^ and inaccurate positioning of the shearing tool away from the adhesive interface might further increase the normal component in the stresses. The contribution of the normal stresses to the adhesive failure is unknown. Therefore, we used 2 different approaches to simulate the adhesive failure in our simulations. The first series of simulations only considered the shear stresses in the contact area (using the contact tangential stress) for determining the glue failure, while the second series of simulations used a combination of the shear and normal stresses in the contact area (additionally considering the contact normal stress) for determining the glue failure.

With both approaches to simulate the adhesive failure, we were able to reproduce the specimen behaviour for all groups as observed in the experiments with regard to the failure force. Even if our approach only works on the loss of contact between the involved components and does not consider potential cracking in the dentin or the composite body, we were able to create a surrogate model that shows the same macroscopic failure behaviour as the specimens in the experiments. In the first simulation series we determined a distinct CTS limit for each of the groups. Unfortunately, even after taking the full spread of experimental measurement results into account, we were not able to derive 1 unique CTS limit value that could be used for all groups. There might be 2 independent reasons for this: the global test parameters ‘specimen dimension’ and ‘shearing tool’ might influence the adhesive failure in the experiment even on the local level; and/or the assumption that only the shear stresses drive the failure in the SBS tests might be false. Without further experimentation, the first point cannot be investigated. The second point can be investigated using our second simulation series.

The observed occurrence of normal stresses in the bonded interface showed that the tensile strength of the adhesive in the normal direction might influence the failure behaviour of the adhesive even in this test, which is designed for shear loading. This shows that our second simulation series might show a better approximation of the failure behaviour of the adhesive than we found in the first series.

In the second simulation series we did not find 1 distinct value for each group, but instead a whole curve of stress limit pairs for each group. This was expected for mathematical reasons, as the underlying mathematical problem (fitting 2 unknown variables to 1 known parameter) is underdetermined. The shape of the curves also fits the mechanical basics: increasing 1 stress limit (either shear or normal stress limit) of a material would increase the failure force, and decreasing the other stress limit would decrease the failure force again. Both processes are continuous (in the mathematical sense), and therefore we can—starting with 1 pair of shear and normal stress limits—vary these limits (increase 1 and decrease the other) and still keep the resulting failure force constant. Comparing the results for all 4 groups, we see no distinct common overlap for all curves, even after taking the standard deviation from the measurements into account. The curves of Micro-WL group in particular show lower stress limits than those of all other groups, which makes it doubtful that 1 common parameter set can be found for all groups. Additionally, it appears that for increasing CNS limits the curves for each geometry converge to the same geometry-specific limits determined in the first series of simulations.

In general, micro shear specimens are always more sensitive to testing variables owing to the technique sensitivity caused by smaller bonding area. Hence, it should be emphasised that despite micro specimens being preferred for shear testing,^7^ they are always more error sensitive in comparison to the macro ones. Errors such as improper adhesive interface engagement or improperly aligned specimen in relation to the shearing tool may lead to dramatic changes in results that will consequently affect the reliability of judgement.

As stated before, the presented numerical model only simulates adhesive failure. We did not simulate the actual crack development in our simulations. Mixed failures during SBS testing (ie, crack propagation continues into the dentin or the composite body) might influence the resulting failure force in either direction. In the previous experimental study, the specimens failed with various failure modes. In the current study, we used the experimental data independent of the failure mode. We might achieve better fitting results by only including measurement data from specimens that only failed in the adhesive layer.

According to the manufacturer of the adhesive used in the experimental part of this study, the shear bond strength of this adhesive is 38 MPa. The stress limits determined in our simulations are much higher and range from 48 MPa for Micro-WL to 127 MPa for Micro-CH. A similar increase can be found between our own experimental results and the numerical results. This seems to be contradicting at first, but the SBS value represents an approximation that uses the full area of specimen base for the calculation. It does not consider that at the time of failure, part of the adhesive has been already detached from the substrate, and the composite body is supported only by a fraction of the original contact area. The value determined in our simulations, on the other hand, is a local parameter that considers each triangle in contact separately: either the glued connection for such triangle is still intact, or the glued connection has failed. In the determination of the SBS value, the overestimation of the surface area still active in the glued connection decreases the calculated stress value artificially. In experimentation, the specimens typically failed in a final forced rupture always occurring in that half of the specimen located away from the shearing tool, which can be identified using, for example, optical microscopy or SEM. In our simulations, the region of the forced rupture was sometimes as low as 25% of the original surface area. This explains the difference between the experimental SBS values and our numerically determined stress limits.

## Conclusions

Within the limitations of this study, the following can be concluded:

• Our proposed numerical method requires individual shear and normal stress limits for each investigated SBS test setup.
• The loading technique and specimen size are important factors influencing shear bond strength.
• For realistic numerical simulation of shear bond strength both, shear and normal stresses should be considered.
• Experimental SBS results that differ in specimen diameter and/or shearing tool cannot be compared directly.

## Conflict of interest

The authors declare no conflict of interests.
