# Erratum to ‘Is Simulation of Glued Contact Sufficient to Simulate Nonlinear Failure Behaviour in Dental Shear Bond Strength Tests?’ [International Dental Journal Volume 75, Issue 3, June 2025, Pages 1746-1758]

**DOI:** 10.1016/j.identj.2025.100915

**Published:** 2025-07-19

**Authors:** Ahmed M. Ismail, Ahmed ElBanna, Tamer M. Nassef, Ludger Keilig, Christoph Bourauel

**Affiliations:** a Oral Technology, University Hospital Bonn, Bonn, Germany; b Biomaterials Department, Faculty of Dentistry, Ain Shams University, Cairo, Egypt; c Computer and Software Engineering Department, Misr University for Science and Technology, Cairo, Egypt; d Department of Prosthodontics, Preclinical Education and Dental Materials Science, School of Dentistry, University Hospital Bonn, Bonn, Germany

The publisher regrets that an important note in the authors’ list had been missed during production. Please see the note below.

The authors Ludger Keilg, and Christoph Bourauel contributed equally to this work.

The publisher would like to apologise for any inconvenience caused.

